# SENP1 Aberrance and Its Linkage to Clinical Features, Adjuvant Regimen, and Prognosis in Patients With Surgical Non-small Cell Lung Cancer Receiving Adjuvant Chemotherapy

**DOI:** 10.3389/fsurg.2021.771785

**Published:** 2022-06-17

**Authors:** Qian Yang, Mengmeng Yang, Jun Zhang, Yuquan Ma

**Affiliations:** Department for Thoracic Surgery, HanDan Central Hospital, Handan, China

**Keywords:** small ubiquitin-like modifier-specific protease 1, surgical non-small cell lung cancer, clinical features, adjuvant chemotherapy regimen, prognosis

## Abstract

**Background:**

Small ubiquitin-like modifier-specific protease 1 (SENP1) plays vital roles in cancer progression and chemoresistance, but its prognostic value in non-small cell lung cancer (NSCLC) is vague. This study aimed to explore the correlation of SENP1 with clinical features, adjuvant chemotherapy regimen, and prognosis in patients with surgical NSCLC receiving adjuvant chemotherapy.

**Methods:**

Tumor and adjacent tissues were collected from 157 patients with surgical NSCLC receiving adjuvant chemotherapy. Meanwhile, tumor tissue and paired adjacent tissue specimens were obtained to evaluate SENP1 protein expression by immunohistochemistry (IHC) assay; among which, 102 pairs were used to detect SENP1 messenger RNA (mRNA) by reverse transcription quantitative PCR.

**Results:**

SENP1 IHC score and SENP1 mRNA expression were increased in tumor tissue than adjacent tissue (*p* < 0.001). Besides, elevated SENP1 IHC score was correlated with > 5 cm tumor size (*p* = 0.045), lymph node metastasis occurrence (*p* = 0.003), and advanced tumor-node-metastasis (TNM) stage (*p* = 0.012); meanwhile, increased SENP1 mRNA expression was associated with histopathological subtype (*p* = 0.011), lymph node metastasis occurrence (*p* = 0.008), and higher TNM stage (*p* = 0.015). Besides, no correlation was found in SENP1 IHC score (*p* = 0.424) or mRNA expression (*p* = 0.927) with specific adjuvant chemotherapy regimen. Additionally, both the SENP1 protein (high) (*p* = 0.003) and mRNA high (*p* = 0.028) were correlated with poor disease-free survival (DFS), while SENP1 protein high was also associated with shorter overall survival (OS) (*p* = 0.029). Furthermore, SENP1 protein (high vs. low) was independently associated with unsatisfying DFS [*p* = 0.009, hazard ratio (HR) = 1.798] and OS (*p* = 0.049, HR = 1.735).

**Conclusion:**

SENP1 may serve as a potential biomarker to improve the management of patients with surgical NSCLC receiving adjuvant chemotherapy.

## Introduction

Lung cancer is a common human cancer with an incidence of nearly two million cases per year and a death number of 1,796,144 in 2020 worldwide (1, 2). Accounting for the majority (80% to 85%) of all the cases of lung cancer, non-small cell lung cancer (NSCLC) is often caused by environmental and genetic factors with cigarette smoking being the major one (3, 4). Over the past two decades, advancements in treating NSCLC have been achieved and adjuvant chemotherapy is recommended for patients with NSCLC at stage II and stage IIIA following surgery (5–7). Although adjuvant chemotherapy improves their survival outcome to some extent, their prognosis remains unsatisfactory due to the high incidence of recurrence (8–10). Therefore, to improve the survival outcome and the management of patients with NSCLC, it is necessary to find out new biomarkers.

Small ubiquitin-like modifier-specific protease 1 (SENP1) is a nuclear protease, which deconjugates small ubiquitin-like modifier (SUMO) ylated proteins (11). It is reported that SENP1 participates in the progression of various cancers. For instance, by mediating deSUMOylation of ubiquitin-conjugating enzyme E2T (UBE2T) and the subsequent protein kinase B (Akt) pathway, SENP1 promotes tumor progression in hepatocellular carcinoma (HCC) (12); meanwhile, by modulating epithelial–mesenchymal transition (EMT), SENP1 plays a vital role in invasion and migration of HCC cells (13); by regulating phosphatase and tensin homolog (PTEN) stability, SENP1 facilitates prostate cancer progression (14); SENP1 also involves in irinotecan resistance in colon cancer (15). Besides, in terms of NSCLC, it is suggested that overexpression of SENP1 in NSCLC relates to chemotherapy resistance. For example, one study suggests that SENP1 expression in tumor is negatively correlated with treatment response in patients with NSCLC (16); another study reports that SENP1 is a potential predictive factor for chemosensitivity in patients with NSCLC (17). Based on the above information, we hypothesized that SENP1 might be a potential biomarker for patients with surgical NSCLC receiving adjuvant chemotherapy. However, no previous study investigated this issue.

Therefore, this study measured the expression of SENP1 by immunohistochemistry (IHC) assay and reverse transcription quantitative PCR (RT-qPCR) detection, with the objective to explore the correlation of SENP1 expression with clinical features, chemotherapy regimen, and prognosis in patients with surgical NSCLC receiving adjuvant chemotherapy.

## Methods

### Patients

This retrospective study reviewed 157 patients with NSCLC who received surgical resection and adjuvant chemotherapy in our hospital between January 2016 and December 2019. The screening criteria were: (i) diagnosed as primary NSCLC according to the European Society for Medical Oncology (ESMO) clinical recommendation (18); (ii) aged over 18 years; (iii) tumor-node-metastasis (TNM) stages II-III; (iv) the Eastern Cooperative Oncology Group Performance Status (ECOG PS) scores 0–1; (v) underwent NSCLC surgical resection and adjuvant chemotherapy; and (vi) had available specimens to perform IHC assay. The exclusion criteria were: (i) had history of other cancers or malignancies at diagnosis; (ii) underwent chemotherapy or radiotherapy before surgical resection; and (iii) without complete clinical characteristics and survival data for analysis. This study was approved by the Institutional Review Board.

### Collection of Clinical Data

The following clinical characteristics of all the patients were recorded in this study: age, gender, smoking, drinking, hypertension, hyperlipidemia, diabetes, histopathological subtype, differentiation, tumor size, lymph node metastasis, TNM stage, the ECOG PS score, carcinoembryonic antigen (CEA), and cancer antigen 125 (CA125). Besides, the adjuvant chemotherapy regimen was also recorded, which included vinorelbine + cisplatin (NP), taxol + cisplatin or carboplatin (TP), gemcitabine + cisplatin or carboplatin (GP), and docetaxel + cisplatin or carboplatin (DP). The follow-up was performed by clinic visit or telephone and the final date of follow-up was June 30, 2021. The median follow-up duration was 4.2 years with a 95% CI of 3.8 to 4.6 years, which was estimated using the reverse Kaplan–Meier (KM) method (19). Survival data were collected to assess disease-free survival (DFS) and overall survival (OS).

### Immunohistochemistry Assay

Tumor tissue specimens and paired adjacent tissue specimens of all the patients were used to assess the expression of SENP1 protein by IHC assay. The rabbit monoclonal anti-SENP1 antibody (1:250; Abcam, Waltham, USA) was applied as primary antibody and the goat anti-rabbit immunoglobulin G (IgG) (H&L) (1:2,000; Abcam, Waltham, USA) was applied as secondary antibody (20). Staining images were taken with a light microscope and IHC results were graded according to intensity and density of staining cells: (i) staining intensity: 0 (negative), 1 (weak), 2 (moderate), and 3 (strong) and (ii) staining density: 0 (0%), 1 (1–25%), 2 (26–50%), 3 (51–75%), and 4 (76–100%). The IHC score was calculated by the product of the staining intensity score and staining density score. Based on the IHC score, SENP1 protein expression was classified as high (IHC score > 3) and low (IHC score ≤ 3).

### Reverse Transcription Quantitative PCR Assay

Among 157 patients with NSCLC, a total of 102 tumor tissue and paired adjacent tissue specimens, which were frozen at −80°C, were accessible for RT-qPCR assay to detect the expression of SENP1 messenger RNA (mRNA). The sample was treated by TRIzol™ Reagent (Thermo Fisher Scientific, Waltham, Massachusetts, USA) to extract total RNA, which was then submitted to perform reverse transcription using the iScript™ cDNA Synthesis Kit (with random primer) (Bio-Rad, Hercules, California, USA). After that, qPCR was carried out with the QuantiNova SYBR Green PCR Kit (Qiagen, Duesseldorf, Nordrhein-Westfalen, Germany). Glyceraldehyde 3-phosphate dehydrogenase (GAPDH) was served as reference gene. The quantitative analysis of SENP1 mRNA expression was conducted with the use of 2^−Δ*ΔCt*^ method. Primers were designed referring to a previous study (21). According to the median value (2.593) of SENP1 mRNA in NSCLC tumor, the expression of SENP1 mRNA was classified as high (>2.593) and low (≤ 2.593).

### Statistical Analysis

The SPSS software version 24.0 (IBM Corporation, Armonk, New York, USA) and the GraphPad Prism version 7.01 (GraphPad Software Incorporation, San Diego, California, USA) were used for data analysis and graph plotting, respectively. SENP1 expression between tumor tissue specimens and paired adjacent tissue specimens was compared using the paired samples *t*-test and the Wilcoxon signed-rank test. Variance equality was assessed by F-test. Comparison of SENP1 expression between/among patients with different clinical characteristics was analyzed using the Student's *t*-test, the one-way ANOVA test, the Mann–Whitney *U* test, or the Kruskal–Wallis H rank-sum test. The KM curve was plotted to display survival profile and log-rank test was used to determine accumulating DFS and OS differences between patients. The Cox proportional hazards regression analysis was used for prognostic analysis. Statistical significance was defined as *p* < 0.05.

## Results

### Clinical Characteristics

A total of 157 patients with NSCLC who received surgical resection and adjuvant chemotherapy were included for analyses; the mean age of these patients was 61.4 ± 10.7 years including 29 (18.5%) female patients and 128 (81.5%) male patients. Their clinical characteristics are shown in Table 1. With respect to the histopathological subtype, there were 84 (53.5%), 54 (34.4%), and 19 (12.1%) patients with adenocarcinoma, squamous cell carcinoma, and adenosquamous carcinoma, respectively. Meanwhile, 37 (23.6%) patients had well differentiation, 68 (43.3%) patients had moderate differentiation, and 52 (33.1%) patients had poor differentiation. Besides, there were 90 (57.3%) and 67 (42.7%) at TNM stages II and III, respectively. Concerning adjuvant chemotherapy regimen, the number of patients who received NP, TP, GP, and DP was 79 (50.3%), 24 (15.3%), 25 (15.9%), and 29 (18.5%), respectively.

**Table 1:** Clinical characteristics.

**Items**	**NSCLC patients (*N* = 157)**
Age (years), mean ± SD	61.4 ± 10.7
Gender, No. (%)	
Female	29 (18.5)
Male	128 (81.5)
Smoking, No. (%)	90 (57.3)
Drinking, No. (%)	60 (38.2)
Hypertension, No. (%)	47 (29.9)
Hyperlipidemia, No. (%)	48 (30.6)
Diabetes, No. (%)	22 (14.0)
Histopathological subtype, No. (%)	
ADC	84 (53.5)
SCC	54 (34.4)
ASC	19 (12.1)
Differentiation, No. (%)	
Well	37 (23.6)
Moderate	68 (43.3)
Poor	52 (33.1)
Tumor size (cm), median (IQR)	6.0 (4.0–8.0)
Lymph node metastasis, No. (%)	70 (44.6)
TNM stage, No. (%)	
II	90 (57.3)
III	67 (42.7)
ECOG PS score, No. (%)	
0	122 (77.7)
1	35 (22.3)
CEA (ng/mL), median (IQR)	5.6 (2.8–33.4)
CA125 (U/mL), median (IQR)	34.5 (14.0–77.9)
Adjuvant chemotherapy regimen, No. (%)	
NP	79 (50.3)
TP	24 (15.3)
GP	25 (15.9)
DP	29 (18.5)

*NSCLC, non-small cell lung cancer; ADC, adenocarcinoma; SCC, squamous cell carcinoma; ASC, adenosquamous carcinoma; IQR, interquartile range; TNM, tumor-node-metastasis; ECOG PS, Eastern Cooperative Oncology Group Performance Status; CEA, carcinoembryonic antigen; CA125, cancer antigen 125; NP, vinorelbine + cisplatin; TP, taxol + cisplatin or carboplatin; GP, gemcitabine + cisplatin or carboplatin; DP, docetaxel+ cisplatin or carboplatin*.

### Small Ubiquitin-Like Modifier-Specific Protease 1 Expression in the Tumor Tissue and Adjacent Tissue

**Figure 1:**
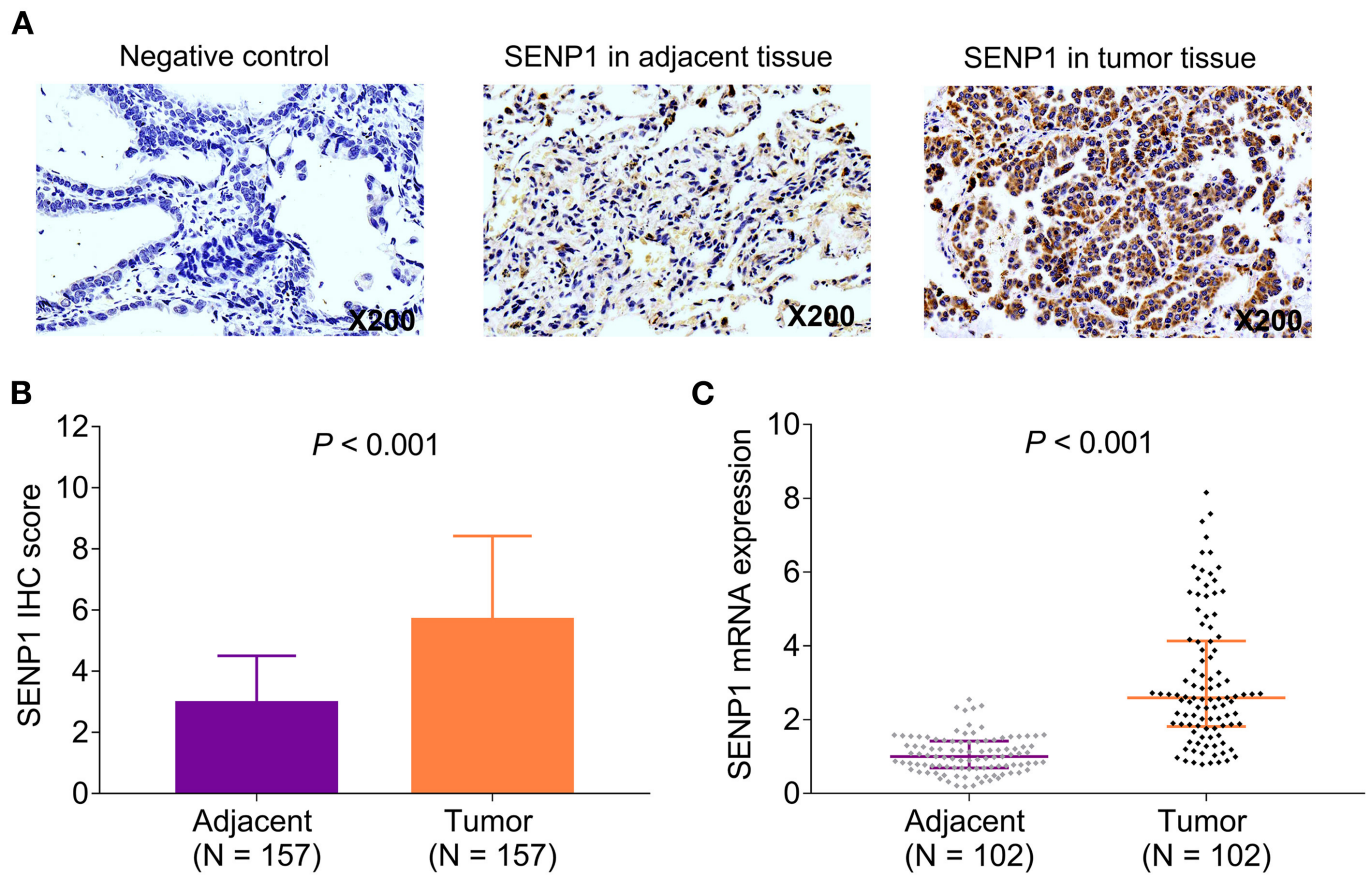
SENP1 expression in patients with surgical NSCLC receiving adjuvant chemotherapy. Examples of SENP1 IHC staining in negative control, adjacent tissues, and tumor tissues **(A)** comparison of SENP1 IHC score **(B)** and SENP1 mRNA expression **(C)** between the tumor tissues and the adjacent tissues. SENP1, small ubiquitin-like modifier (SUMO)-specific protease 1; IHC, immunohistochemistry; NSCLC, non-small cell lung cancer.

The expression of SENP1 protein was assessed in the tumor tissue and paired adjacent tissue by IHC assay (Figure 1A). Meanwhile, SENP1 IHC score was higher in the tumor tissue than in the adjacent tissue (*n* = 157) (*p* < 0.001) (Figure 1B); SENP1 mRNA expression in the tumor tissue was also elevated than that in the adjacent tissue (*n* = 102) (*p* < 0.001) (Figure 1C).

### Correlation Between SENP1 Expression and Clinical Features

Elevated SENP1 IHC score was correlated with > 5 cm tumor size (*p* = 0.045), the occurrence of lymph node metastasis (*p* = 0.003), and more advanced TNM stage (*p* = 0.012). However, no correlation was found in the SENP1 IHC score with other clinical features (*p* > 0.05). In addition, increased SENP1 mRNA expression was associated with histopathological subtype (*p* = 0.011), the occurrence of lymph node metastasis (*p* = 0.008), and higher TNM stage (*p* = 0.015). However, no association of SENP1 mRNA expression with other clinical features was observed (*p* > 0.05) (Table 2).

**Table 2:** Correlation between SENP1 expression and clinical characteristics.

**Items**	**SENP1 IHC score**		**SENP1 mRNA expression**	
	**Mean ±SD**	***P*-value**	**Median (IQR)**	***P*-value**
Age		0.231		0.272
≤ 60 years	5.3 ± 2.8		2.492 (1.281–4.122)	
> 60 years	5.9 ± 2.7		2.669 (1.871–4.171)	
Gender		0.337		0.338
Female	6.1 ± 3.1		2.846 (1.836–5.429)	
Male	5.6 ± 2.7		2.566 (1.756–3.878)	
Smoking		0.104		0.768
No	5.2 ± 2.5		2.570 (1.315–4.437)	
Yes	6.0 ± 2.9		2.649 (1.879–3.392)	
Drinking		0.108		0.407
No	5.4 ± 2.5		2.680 (1.815–4.189)	
Yes	6.1 ± 3.1		2.435 (1.713–3.480)	
Hypertension		0.282		0.298
No	5.5 ± 2.6		2.629 (1.794–3.641)	
Yes	6.0 ± 3.1		2.553 (1.814–5.796)	
Hyperlipidemia		0.732		0.971
No	5.6 ± 2.7		2.600 (1.845–3.831)	
Yes	5.8 ± 2.9		2.565 (1.648–4.276)	
Diabetes		0.061		0.355
No	5.5 ± 2.7		2.577 (1.835–3.880)	
Yes	6.7 ± 3.0		3.722 (1.521–5.737)	
Histopathological subtype		0.785		**0.011**
ADC	5.8 ± 2.8		2.698 (2.021–4.694)	
SCC	5.4 ± 2.9		1.672 (1.095–2.933)	
ASC	5.7 ± 2.4		2.697 (2.118–3.891)	
Differentiation		0.190		0.189
Well	5.4 ± 2.8		1.958 (1.283–2.663)	
Moderate	5.5 ± 2.6		3.101 (1.820–5.442)	
Poor	6.1 ± 2.9		2.566 (1.962–3.445)	
Tumor size		**0.045**		0.437
≤ 5 cm	5.1 ± 2.2		2.582 (1.310–4.119)	
> 5 cm	6.0 ± 3.0		2.608 (1.896–4.189)	
Lymph node metastasis		**0.003**		**0.008**
No	5.1 ± 2.5		2.318 (1.519–3.053)	
Yes	6.4 ± 3.0		3.055 (2.167–5.349)	
TNM stage		**0.012**		**0.015**
II	5.2 ± 2.3		2.318 (1.376–3.300)	
III	6.3 ± 3.2		2.704 (2.251–4.920)	
ECOG PS score		0.269		0.056
0	5.5 ± 2.7		2.442 (1.693–4.052)	
1	6.1 ± 3.0		2.894 (2.560–5.460)	
CEA		0.852		0.976
Normal (≤ 5 ng/ml)	5.6 ± 2.6		2.598 (1.574–4.243)	
Abnormal (> 5 ng/ml)	5.7 ± 2.9		2.553 (1.871–3.885)	
CA125		0.732		0.954
Normal (≤ 35 U/ml)	5.6 ± 2.6		2.548 (1.862–4.501)	
Abnormal (> 35 U/ml)	5.7 ± 2.9		2.684 (1.765–3.325)	

*SENP1, small ubiquitin-like modifier (SUMO)-specific protease 1; IQR, interquartile range; ADC, adenocarcinoma; SCC, squamous cell carcinoma; ASC, adenosquamous carcinoma; TNM, tumor-node-metastasis; ECOG PS, Eastern Cooperative Oncology Group Performance Status; CEA, carcinoembryonic antigen; CA125, cancer antigen 125*.

*The bold value indicates statistical significance*.

### Association of SENP1 Expression With Adjuvant Chemotherapy Regimen

**Figure 2:**
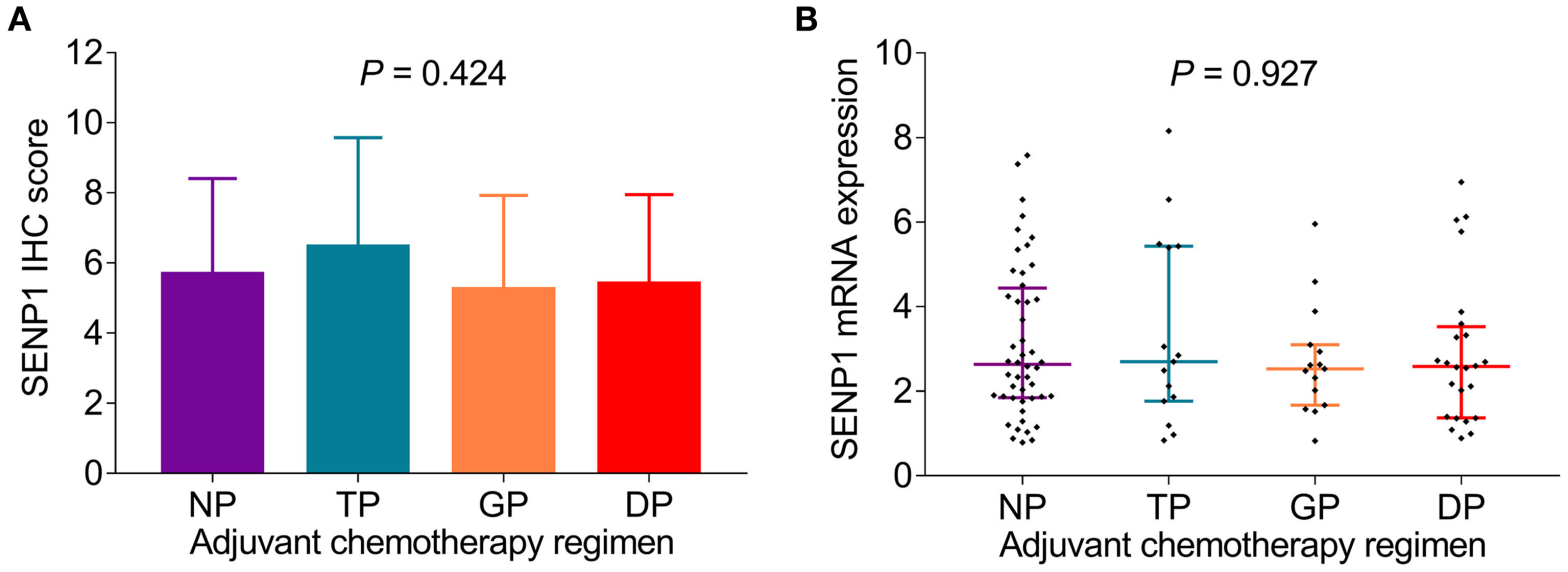
Comparison of SENP1 expression among patients with surgical NSCLC receiving different adjuvant chemotherapy regimens. Comparison of SENP1 IHC score **(A)** and SENP1 mRNA expression **(B)** among patients with NSCLC who received NP, TP, GP, and DP. SENP1, small ubiquitin-like modifier (SUMO)-specific protease 1; IHC, immunohistochemistry; NP, vinorelbine + cisplatin; TP, taxol + cisplatin or carboplatin; GP, gemcitabine + cisplatin or carboplatin; DP, docetaxel + cisplatin or carboplatin; NSCLC, non-small cell lung cancer.

Adjuvant chemotherapy regimen was recorded in this study and analysis of the correlation of SENP1 expression with adjuvant chemotherapy regimen was conducted, which discovered that both the SENP1 IHC score (*p* = 0.424) and SENP1 mRNA expression (*p* = 0.927) showed no correlation with adjuvant chemotherapy regimen (Figures 2A,B).

### Correlation of SENP1 Expression With Accumulating DFS

**Figure 3:**
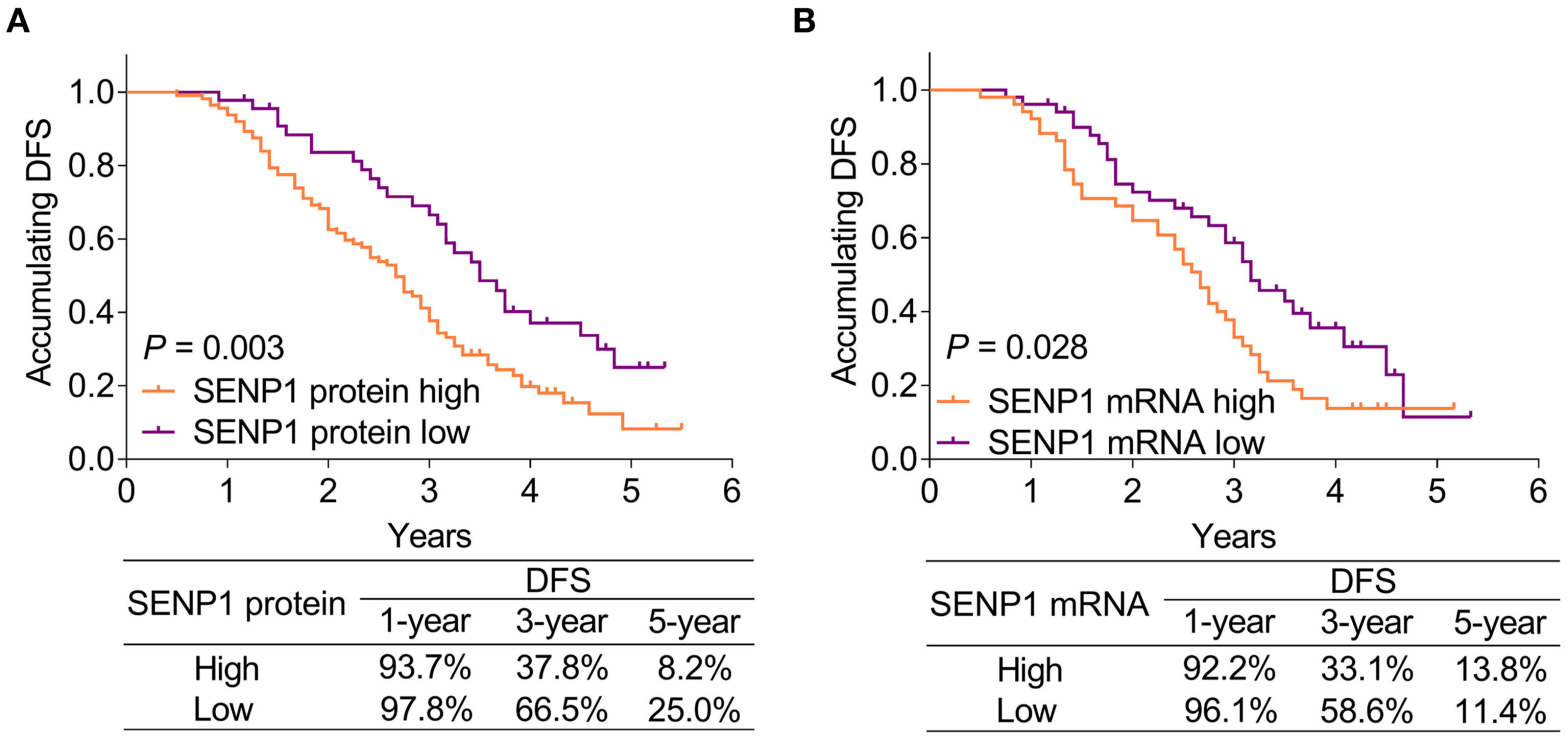
Comparison of accumulating DFS between patients with surgical NSCLC with different SENP1 expressions. Comparison of accumulating DFS between patients with surgical NSCLC with SENP1 protein high and SENP1 protein low **(A)** and comparison of accumulating DFS between patients with surgical NSCLC with SENP1 mRNA high and SENP1 mRNA low **(B)**. SENP1, small ubiquitin-like modifier (SUMO)-specific protease 1; DFS, disease-free survival; NSCLC, non-small cell lung cancer.

Small ubiquitin-like modifier-specific protease 1 protein high was correlated with poor accumulating DFS (*p* = 0.003). Meanwhile, the 1-year, 3-year, and 5-year DFS rates of patients with SENP1 protein high were 93.7, 37.8, and 8.2%, respectively, while those of patients with SENP1 protein low were 97.8, 66.5, and 25.0%, respectively (Figure 3A). Besides, SENP1 mRNA high was also associated with worse accumulating DFS (*p* = 0.028) and the 1-year, 3-year, and 5-year DFS rate in patients with SENP1 mRNA high were 92.2, 33.1, and 13.8%, respectively, while those in patients with SENP1 mRNA low were 96.1, 58.6, and 11.4%, respectively (Figure 3B).

Additionally, according to the univariate Cox proportional hazards regression analysis, SENP1 protein (high vs. low) [*p* = 0.004, hazard ratio (HR) = 1.889], SENP1 mRNA (high vs. low) (*p* = 0.032, HR = 1.676), poor differentiation (*p* = 0.002, HR = 1.524), tumor size (>5 vs. ≤ 5 cm) (*p* = 0.020, HR = 1.603), lymph node metastasis (yes vs. no) (*p* = 0.007, HR = 1.679), TNM stage (III vs. II) (*p* = 0.001, HR = 1.874), and CA125 (>35 vs. ≤ 35 U/ml) (*p* = 0.034, HR = 1.500) were correlated with shorter accumulating DFS. Furthermore, the multivariate Cox proportional hazards regression analysis showed that SENP1 protein (high vs. low) (*p* = 0.009, HR = 1.798), age (>60 vs. ≤ 60 years) (*p* = 0.019, HR = 1.617), poor differentiation (*p* = 0.011, HR = 1.422), and TNM stage (III vs. II) (*p* = 0.002, HR = 1.811) were independently associated with unsatisfying accumulating DFS (Table 3).

**Table 3:** The Cox proportional hazards regression analysis for DFS.

**Items**	***P-*value**	**HR**	**95% CI**	
			**Lower**	**Upper**
**Univariate Cox proportional hazards regression analysis**				
SENP1 protein (high vs. low)	**0.004**	1.889	1.222	2.921
SENP1 mRNA (high vs. low)	**0.032**	1.676	1.044	2.691
Age (> 60 vs. ≤ 60 years)	0.052	1.478	0.996	2.195
Gender (male vs. female)	0.412	1.231	0.750	2.022
Smoking (yes vs. no)	0.281	1.233	0.843	1.805
Drinking (yes vs. no)	0.414	0.851	0.577	1.254
Hypertension (yes vs. no)	0.762	0.939	0.623	1.414
Hyperlipidemia (yes vs. no)	0.839	1.043	0.694	1.568
Diabetes (yes vs. no)	0.813	1.064	0.635	1.785
**Histopathological subtype**				
ASC	Reference			
ADC	0.710	0.903	0.527	1.548
SCC	0.104	0.613	0.339	1.106
Poor differentiation	**0.002**	1.524	1.169	1.987
Tumor size (> 5 vs. ≤ 5 cm)	**0.020**	1.603	1.077	2.387
Lymph node metastasis (yes vs. no)	**0.007**	1.679	1.152	2.446
TNM stage (III vs, II)	**0.001**	1.874	1.286	2.730
ECOG PS score (1 vs. 0)	0.193	1.337	0.864	2.070
CEA (> 5 vs. ≤ 5 ng/ml)	0.155	1.317	0.901	1.924
CA125 (> 35 vs. ≤ 35 U/ml)	**0.034**	1.500	1.031	2.182
**Adjuvant chemotherapy regimen**				
DP	Reference			
NP	0.530	0.848	0.506	1.420
TP	0.955	1.019	0.528	1.969
GP	0.391	1.305	0.710	2.399
**Forward stepwise multivariate Cox proportional hazards regression analysis**				
SENP1 protein (high vs. low)	**0.009**	1.798	1.161	2.783
Age (> 60 vs. ≤ 60 years)	**0.019**	1.617	1.081	2.419
Poor differentiation	**0.011**	1.422	1.085	1.864
TNM stage (III vs, II)	**0.002**	1.811	1.235	2.654

*DFS, disease-free survival; HR, hazard ratio; SENP1, small ubiquitin-like modifier (SUMO)-specific protease 1; ASC, adenosquamous carcinoma; ADC, adenocarcinoma; SCC, squamous cell carcinoma; TNM, tumor-node-metastasis; ECOG PS, Eastern Cooperative Oncology Group Performance Status; CEA, carcinoembryonic antigen; CA125, cancer antigen 125*.

*The bold value indicates statistical significance*.

### Association of SENP1 Expression With Accumulating OS

**Figure 4:**
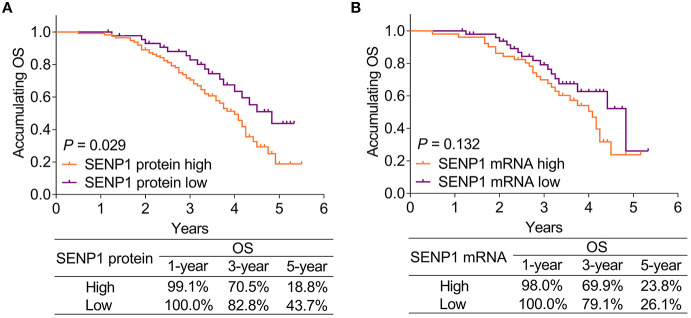
Comparison of accumulating OS between patients with surgical NSCLC with different SENP1 expressions. Comparison of accumulating OS between patients with surgical NSCLC with SENP1 protein high and SENP1 protein low **(A)** and comparison of accumulating OS between patients with surgical NSCLC with SENP1 mRNA high and SENP1 mRNA low **(B)**. SENP1, small ubiquitin-like modifier (SUMO)-specific protease 1; OS, overall survival; NSCLC, non-small cell lung cancer.

Small ubiquitin-like modifier-specific protease 1 protein high was associated with shorter accumulating OS (*p* = 0.029). In patients with SENP1 protein high, the 1-year, 3-year, and 5-year OS rates were 99.1, 70.5, and 18.8%, respectively, while those in patients with SENP1 protein low were 100.0, 82.8, and 43.7%, respectively (Figure 4A). However, no correlation was found in SENP1 mRNA with accumulating OS (*p* = 0.132). Additionally, the 1-year, 3-year, and 5-year OS rates of patients with SENP1 mRNA high were 98.0, 69.9, and 23.8%, respectively, while those of patients with SENP1 mRNA low were 100.0, 79.1, and 26.1%, respectively (Figure 4B).

Moreover, the univariate Cox proportional hazards regression analysis suggested that SENP1 protein (high vs. low) (*p* = 0.033, HR = 1.816), age (>60 vs. ≤ 60 years) (*p* = 0.014, HR = 1.887), poor differentiation (*p* < 0.001, HR = 1.868), tumor size (>5 vs. ≤ 5 cm) (*p* = 0.002, HR = 2.285), lymph node metastasis (yes vs. no) (*p* < 0.001, HR = 2.412), TNM stage (III vs. II) (*p* < 0.001, HR = 2.542), and CA125 (>35 vs. ≤ 35 U/ml) (*p* = 0.032, HR = 1.671) were correlated with unsatisfying accumulating OS. Furthermore, the multivariate Cox proportional hazards regression analysis revealed that SENP1 protein (high vs. low) (*p* = 0.049, HR = 1.735), age (>60 vs. ≤ 60 years) (*p* = 0.005, HR = 2.119), poor differentiation (*p* = 0.001, HR = 1.843), and TNM stage (III vs. II) (*p* < 0.001, HR = 2.415) were independently associated with poor accumulating OS (Table 4).

**Table 4:** The Cox proportional hazards regression analysis for OS.

**Items**	***P-*value**	**HR**	**95% CI**	
			**Lower**	**Upper**
**Univariate Cox proportional hazards regression analysis**				
SENP1 protein (high vs. low)	**0.033**	1.816	1.049	3.141
SENP1 mRNA (high vs. low)	0.138	1.593	0.861	2.947
Age (> 60 vs. ≤ 60 years)	**0.014**	1.887	1.138	3.128
Gender (male vs. female)	0.510	1.231	0.663	2.288
Smoking (yes vs. no)	0.506	1.173	0.733	1.880
Drinking (yes vs. no)	0.834	1.052	0.656	1.687
Hypertension (yes vs. no)	0.495	0.833	0.493	1.408
Hyperlipidemia (yes vs. no)	0.144	1.432	0.885	2.317
Diabetes (yes vs. no)	0.533	0.801	0.398	1.610
**Histopathological subtype**				
ASC	Reference			
ADC	0.523	0.809	0.422	1.551
SCC	0.373	0.727	0.361	1.465
Poor differentiation	**<0.001**	1.868	1.318	2.646
Tumor size (> 5 vs. ≤ 5 cm)	**0.002**	2.285	1.351	3.864
Lymph node metastasis (yes vs. no)	**<0.001**	2.412	1.489	3.907
TNM stage (III vs, II)	**<0.001**	2.542	1.592	4.059
ECOG PS score (1 vs. 0)	0.504	1.193	0.712	1.999
CEA (> 5 vs. ≤ 5 ng/ml)	0.215	1.345	0.841	2.151
CA125 (> 35 vs. ≤ 35 U/ml)	**0.032**	1.671	1.045	2.674
**Adjuvant chemotherapy regimen**				
DP	Reference			
NP	0.834	1.075	0.547	2.112
TP	0.553	1.281	0.565	2.906
GP	0.658	1.200	0.535	2.694
**Forward stepwise multivariate Cox proportional hazards regression analysis**				
SENP1 protein (high vs. low)	**0.049**	1.735	1.003	3.000
Age (> 60 vs. ≤ 60 years)	**0.005**	2.119	1.262	3.557
Poor differentiation	**0.001**	1.843	1.289	2.635
TNM stage (III vs, II)	**<0.001**	2.415	1.504	3.879

*OS, overall survival; HR, hazard ratio; SENP1, small ubiquitin-like modifier (SUMO)-specific protease 1; ASC, adenosquamous carcinoma; ADC, adenocarcinoma; SCC, squamous cell carcinoma; TNM, tumor-node-metastasis; ECOG PS, Eastern Cooperative Oncology Group Performance Status; CEA, carcinoembryonic antigen; CA125, cancer antigen 125*.

*The bold value indicates statistical significance*.

## Discussion

With respect to the SENP1 expression in cancer tissues and adjacent tissues, it is suggested that SENP1 upregulates in pancreatic ductal adenocarcinoma tissues than in adjacent tissues (22). Additionally, the expression of SENP1 is higher in tumor tissues than paracarcinoma tissues in patients with HCC (12). This study found that SENP1 expression was increased in NSCLC tumor tissues than adjacent tissues in patients with surgical NSCLC receiving adjuvant chemotherapy. A possible reason could be that: SENP1 reflected the higher proliferation rate of cells; meanwhile, the proliferation rate in NSCLC cells in the tumor tissue was increased than that in the adjacent tissue cells. Thus, SENP1 expression was higher in NSCLC tumor tissues compared with adjacent tissues in patients with surgical NSCLC receiving adjuvant chemotherapy.

In terms of the correlation of SENP1 with clinical features, a previous study shows that SENP1 expression positively correlates with lymph node metastasis and TNM stage in patients with pancreatic cancer (22). Another study suggests that plasma exosome-derived SENP1 associates with higher tumor diameter and tumor stage in patients with osteosarcoma (23). Besides, SENP1 overexpression is correlated with moderate and low differentiation of NSCLC tumors (11). In this study, we observed that in patients with surgical NSCLC receiving adjuvant chemotherapy, SENP1 expression was correlated with larger tumor size, histopathological subtype, the occurrence of lymph node metastasis, and higher TNM stage. Possible explanations could be that: (1) through the hypoxia-induced factor-1α (HIF-1α) signaling pathway, SENP1 could promote the proliferation of NSCLC cancer cells, resulting in larger tumor size; (2) SENP1 could regulate matrix metalloproteinase-9 (MMP-9) to promote NSCLC cancer metastasis; meanwhile, SENP1 might enhance NSCLC cell invasive ability via modulating epithelial–mesenchymal transition marked genes, which contributed to the occurrence of lymph node metastasis (22, 24). Thus, SENP1 expression was associated with larger tumor size and occurrence of lymph node metastasis (17); and (3) SENP1 expression was correlated with larger tumor size and lymph node metastasis, which were features of more advanced TNM stage. Therefore, SENP1 expression was associated with higher TNM stage (25).

Moreover, no correlation was found in SENP1 expression with adjuvant chemotherapy regimen in this study, which could be explained by that: adjuvant chemotherapy regimen was applied after surgical resection and the sample for analyzing was collected before but not during or after adjuvant chemotherapy, thus no correlation in SENP1 expression with adjuvant chemotherapy regimen was found.

Concerning the association of SENP1 expression with prognosis in patients with cancer, higher plasma exosome-derived SENP1 correlates with worse DFS and OS in patients with osteosarcoma (23); additionally, SENP1 overexpression independently correlates with poor prognosis in patients with NSCLC (11). This study discovered that both the SENP1 protein high and SENP1 mRNA high were correlated with poor accumulating DFS, while SENP1 protein high was also associated with shorter accumulating OS in patients with surgical NSCLC receiving adjuvant chemotherapy. Meanwhile, SENP1 protein (high vs. low) was an independent risk factor for unsatisfying accumulating DFS and OS. The explanation could be that: (1) as mentioned earlier, SENP1 was related to chemotherapy resistance in patients with surgical NSCLC receiving adjuvant chemotherapy (11), further causing unsatisfying DFS and OS and (2) SENP1 expression was correlated with the occurrence of lymph node metastasis and higher TNM stage (as mentioned above), which could indirectly cause poor prognosis.

Except for the above discussion and explanation, detection of SENP1 might have the following clinical implication: SENP1 might serve as an indicator for tumor characteristics and prognosis in NSCLC, which could further improve the management of patients with NSCLC. Furthermore, the detection of SENP1 might influence the choice of different therapies and serve as a decision-making factor in the choice or the change of therapy. However, these descriptions needed a number of multicenter prospective studies with larger sample size to validate further findings.

Although a lot of findings were identified, there were still some limitations in this study. First, this study had a relatively small sample size, which might cause low statistical power; secondly, this study did not investigate the underlying mechanism of SENP1 in NSCLC progression and chemoresistance. Therefore, further *in-vivo* and *in-vitro* experiments were needed; third, although this study had a 5-year follow-up duration, longer follow-up could be conducted in the future to investigate the long-term prognostic effect of SENP1 in patients with surgical NSCLC receiving adjuvant chemotherapy; fourth, blood samples of patients with surgical NSCLC receiving adjuvant chemotherapy might be collected in the future study to compare and analyze the changes of SENP1 protein before and after adjuvant chemotherapy; fifth, since this was a retrospective study, its evidence-based medicine was of low value, thus a prospective study might be further performed to validate the findings.

## Conclusion

Small ubiquitin-like modifier-specific protease 1 overexpression correlates with larger tumor size, lymph node metastasis, higher TNM stage, as well as shorter DFS and OS in patients with surgical NSCLC receiving adjuvant chemotherapy.

## Data Availability Statement

The original contributions presented in the study are included in the article/supplementary material, further inquiries can be directed to the corresponding author/s.

## Ethics Statement

The studies involving human participants were reviewed and approved by HanDan Central Hospital. The patients/participants provided their written informed consent to participate in this study.

## Author Contributions

QY and YM contribute to the conception, design, data analysis, and interpretation. QY contributes to the administrative support. QY, MY, JZ, and YM contribute to the provision of study materials or patients. QY, MY, and JZ contribute to the collection and assembly of data. All the authors involved in writing of manuscript and submitted the final approval of the manuscript.

## Conflict of Interest

The authors declare that the research was conducted in the absence of any commercial or financial relationships that could be construed as a potential conflict of interest.

## Publisher's Note

All claims expressed in this article are solely those of the authors and do not necessarily represent those of their affiliated organizations, or those of the publisher, the editors and the reviewers. Any product that may be evaluated in this article, or claim that may be made by its manufacturer, is not guaranteed or endorsed by the publisher.

## References

[1] Suster DI Mino-Kenudson M. Molecular pathology of primary non-small cell lung cancer. Arch Med Res. (2020) 51:784–98. 10.1016/j.arcmed.2020.08.004 32873398

[2] Thai AA Solomon BJ Sequist LV Gainor JF Heist RS. Lung cancer. Lancet. (2021) 398:535–54. 10.1016/S0140-6736(21)00312-3 34273294

[3] Zappa C Mousa SA. Non-small cell lung cancer: current treatment and future advances. Transl Lung Cancer Res. (2016) 5:288–300. 10.21037/tlcr.2016.06.07 27413711 PMC4931124

[4] Khan S Ali S Muhammad. Exhaustive review on lung cancers: novel technologies. Curr Med Imaging Rev. (2019) 15:873–83. 10.2174/1573405615666181128124528 32013812

[5] Kris MG Gaspar LE Chaft JE Kennedy EB Azzoli CG Ellis PM. Adjuvant systemic therapy and adjuvant radiation therapy for stage I to IIIA completely resected non-small-cell lung cancers: American Society of Clinical Oncology/Cancer Care Ontario clinical practice guideline update. J Clin Oncol. (2017) 35:2960–74. 10.1200/JCO.2017.72.4401 28437162

[6] Herbst RS Morgensztern D Boshoff C. The biology and management of non-small cell lung cancer. Nature. (2018) 553:446–54. 10.1038/nature25183 29364287

[7] Valladares BT Crespo PC Herranz UA Caamano AG. Adjuvant treatment in lung cancer. J Clin Transl Res. (2021) 7:175–84. 10.18053/jctres.07.202102.012 34104820 PMC8177857

[8] Santarpia M Rolfo C Peters GJ Leon LG Giovannetti E. On the pharmacogenetics of non-small cell lung cancer treatment. Expert Opin Drug Metab Toxicol. (2016) 12:307–17. 10.1517/17425255.2016.1141894 26761638

[9] Arbour KC Riely GJ. Systemic therapy for locally advanced and metastatic non-small cell lung cancer: a review. JAMA. (2019) 322:764–74. 10.1001/jama.2019.11058 31454018

[10] Mulherkar R Grewal AS Berman AT. Emerging role of immunotherapy in locally advanced non-small cell lung cancer. Clin Adv Hematol Oncol. (2020) 18:212–17. 32628649

[11] Zuo Y Cheng JK. Small ubiquitin-like modifier protein-specific protease 1 and prostate cancer. Asian J Androl. (2009) 11:36–8. 10.1038/aja.2008.45 19050680 PMC3735207

[12] Tao Y Li R Shen C Li J Zhang Q Ma Z. SENP1 is a crucial promotor for hepatocellular carcinoma through deSUMOylation of UBE2T. Aging. (2020) 12:1563–76. 10.18632/aging.102700 31969492 PMC7053586

[13] Zhang W Sun H Shi X Wang H Cui C Xiao F. SENP1 regulates hepatocyte growth factor-induced migration and epithelial-mesenchymal transition of hepatocellular carcinoma. Tumour Biol. (2016) 37:7741–8. 10.1007/s13277-015-4406-y 26695141

[14] Bawa-Khalfe T Yang FM Ritho J Lin HK Cheng J Yeh ET. SENP1 regulates PTEN stability to dictate prostate cancer development. Oncotarget. (2017) 8:17651–64. 10.18632/oncotarget.13283 27852060 PMC5392276

[15] Chen MC Nhan DC Hsu CH Wang TF Li CC Ho TJ. SENP1 participates in Irinotecan resistance in human colon cancer cells. J Cell Biochem. (2021) 122:1277–94. 10.1002/jcb.29946 34037277

[16] Liu K Zhang J Wang H. Small ubiquitin-like modifier/sentrin-specific peptidase 1 associates with chemotherapy and is a risk factor for poor prognosis of non-small cell lung cancer. J Clin Lab Anal. (2018) 32:e22611. 10.1002/jcla.22611 30043429 PMC6817010

[17] Mu J Zuo Y Yang W Chen Z Liu Z Tu J. Over-expression of small ubiquitin-like modifier proteases 1 predicts chemo-sensitivity and poor survival in non-small cell lung cancer. Chin Med J. (2014) 127:4060–5. 10.3760/cma.j.issn.0366-6999.20141013 25430449

[18] D'Addario G Felip E Group EGW. Non-small-cell lung cancer: ESMO clinical recommendations for diagnosis, treatment and follow-up. Ann Oncol. (2009) 20 Suppl 4:68–70. 10.1093/annonc/mdp132 19454467

[19] Schemper M Smith TL. A note on quantifying follow-up in studies of failure time. Control Clin Trials. (1996) 17:343–6. 10.1016/0197-2456(96)00075-X 8889347

[20] Lin X Wang Y Jiang Y Xu M Pang Q Sun J. Sumoylation enhances the activity of the TGF-beta/SMAD and HIF-1 signaling pathways in keloids. Life Sci. (2020) 255:117859. 10.1016/j.lfs.2020.117859 32474020

[21] Li T Huang S Dong M Gui Y Wu D. Prognostic impact of SUMO-specific protease 1 (SENP1) in prostate cancer patients undergoing radical prostatectomy. Urol Oncol. (2013) 31:1539–45. 10.1016/j.urolonc.2012.03.007 23089540

[22] Ma C Wu B Huang X Yuan Z Nong K Dong B. SUMO-specific protease 1 regulates pancreatic cancer cell proliferation and invasion by targeting MMP-9. Tumour Biol. (2014) 35:12729–35. 10.1007/s13277-014-2598-1 25217324

[23] Wang L Wu J Song S Chen H Hu Y Xu B. Plasma exosome-derived sentrin SUMO-specific protease 1: a prognostic biomarker in patients with osteosarcoma. Front Oncol. (2021) 11:625109. 10.3389/fonc.2021.625109 33791211 PMC8006461

[24] Wang X Liang X Liang H Wang B. SENP1/HIF-1alpha feedback loop modulates hypoxia-induced cell proliferation, invasion, and EMT in human osteosarcoma cells. J Cell Biochem. (2018) 119:1819–26. 10.1002/jcb.26342 28796315

[25] Woodard GA Jones KD Jablons DM. Lung cancer staging and prognosis. Cancer Treat Res. (2016) 170:47–75. 10.1007/978-3-319-40389-2_3 27535389

